# Assessing the affective component of pain, and the efficacy of pain control, using conditioned place aversion in calves

**DOI:** 10.1098/rsbl.2019.0642

**Published:** 2019-10-30

**Authors:** Thomas Ede, Marina A. G. von Keyserlingk, Daniel M. Weary

**Affiliations:** Animal Welfare Program, Faculty of Land and Food Systems, University of British Columbia, Vancouver, British Columbia, Canada

**Keywords:** pain, affective state, animal welfare, cyclooxygenase (COX)

## Abstract

Pain in animals is typically assessed using reflexive and physiological responses. These measures allow inferences regarding nociception but provide little basis for conclusions about the affective component of pain (i.e. how negatively the experience is perceived). Calves routinely undergo painful procedures on commercial farms, including hot-iron disbudding, providing a convenient model to study pain in animals. The aim of this study was to investigate the affective component of post-procedural pain due to hot-iron disbudding, using conditioned place aversion. Calves (*n* = 31) were subjected to two procedures (one bud at a time): one without post-procedural pain control and the other with the use of a nonsteroidal anti-inflammatory drug (either meloxicam (*n* = 16) or ketoprofen (*n* = 15)). All procedures included the use of local anaesthesia (lidocaine). Place conditioning was tested 2 days after the last treatment by allowing calves to freely roam between the pens where they had previously been disbudded. Calves spent more time, and lay down more frequently, in the pen where they received meloxicam compared with the pen where they only received a local block. Surprisingly, calves avoided the pen where they received ketoprofen compared with the control treatment pen. We hypothesize that the shorter duration of action of ketoprofen resulted in increasing pain at the end of the conditioning period, explaining the increased aversion to this treatment. These results illustrate the value of place conditioning paradigms to assess the affective component of pain in animals, and suggest that the animal's evaluation of painful events depends upon the time course of when the pain is experienced.

## 1. Introduction

Many approaches to study animal pain can be found in the literature, most of which rely on either nociceptive processes (e.g. hypersensitivity of injured areas [1]), activation of the hypothalamic–pituitary–adrenal axis (e.g. salivary concentration of cortisol [2]) or indirect measures of activation of the sympathetic nervous system (e.g. heart rate [3]). Such responses reflect the sensory component of pain and do not require processing by the central nervous system. By contrast, the affective component does require central processing as this relates to how negative the experience is perceived to be. Nociception is generally thought to result in inelastic responses (i.e. withdrawal reflex) whereas the affective component of pain contributes to and can be affected by learning [4]. Thus, experimental paradigms based on learned responses (such as preference, motivation and aversion tests) provide a stronger basis to investigate the affective component [5,6].

Although a less common model than rats and mice in the study of pain, dairy calves routinely undergo painful management procedures [7–9]; studying the pain associated with routine procedures avoids the need to cause pain purely for the sake of research. One routine procedure is disbudding, in which horn buds are cauterized using a hot iron (heated to at least 500°C). The resulting burns are painful to calves [10], but the duration and magnitude of the post-procedural pain are still unclear, making it difficult to develop pain mitigation strategies [11,12].

We recently applied the principle of conditioned place aversion to study the affective impact of disbudding in dairy calves: dairy calves avoided a pen where they had been disbudded (with the use of a sedative and local anaesthetic but without post-procedural pain control) compared with a pen where they had only received a sham procedure (i.e. with sedation but without disbudding) [13]. This result indicates that disbudding is a negative affective experience for calves, even when provided with sedation and local anaesthesia to mitigate intra-procedural pain. The observed aversion was likely a consequence of post-procedural pain emerging once the action of the local anaesthetic waned in the hours after disbudding.

Providing calves with a post-procedural analgesic, such as a nonsteroidal anti-inflammatory drug (NSAID), has been shown to reduce reflex and automatic pain responses [14,15], but no study has—to our knowledge—investigated the effect of NSAIDs on the affective component of pain.

The main objective of the current study was to identify whether providing post-procedural analgesics could mitigate the affective component of pain in dairy calves following hot-iron disbudding. We used conditioned place aversion to compare two disbudding procedures: one without post-procedural pain control and one with the use of an NSAID. A secondary objective was to evaluate two different NSAIDs commonly used in veterinary practice to alleviate post-procedural pain: meloxicam and ketoprofen. We predicted that calves would display conditioned place aversion to the environment where they did not receive post-procedural pain control in comparison with the environment where they received either meloxicam or ketoprofen.

## 2. Methods

### (a) Animals

Holstein heifers (*n* = 34) were enrolled at 32 ± 5.9 days of age with a body weight of 68 ± 6.8 kg. Calves were assigned in blocks and balanced to two treatment groups: *meloxicam* and *ketoprofen*. See electronic supplementary material for details.

### (b) Apparatus

**Figure 1.**
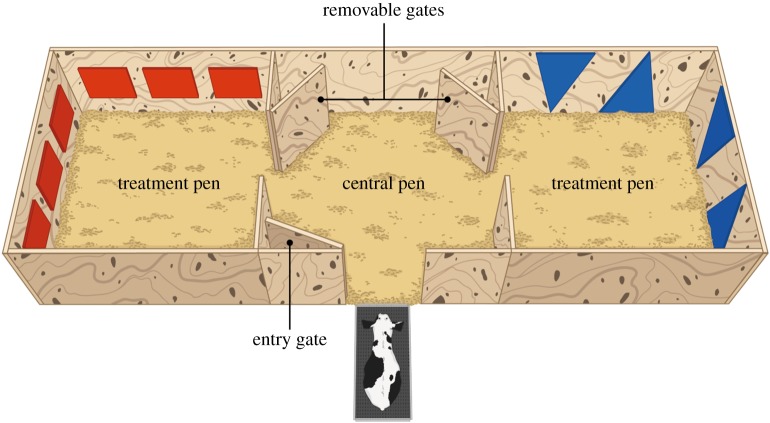
Experimental apparatus. Calves (*n* = 31) received both disbudding procedures (‘Control’: without the use of post-procedural pain mitigation and ‘NSAID’ with the use of either meloxicam (*n* = 16) or ketoprofen (*n* = 15)) and spent the following 6 h in the treatment pens. During test sessions, the removable gates were taken out, allowing the calf to freely roam between pens. (Online version in colour.)

The experimental apparatus was identical to the one used in Ede *et al*. [13]. Briefly, we used a plywood pen (2.1 × 6.0 m), divided into three equal-sized compartments (2.1 × 2.0 m) connected by removable gates (figure 1). Treatment pens were on either side of the central pen; all disbudding procedures took place in the treatment pens. The walls of each treatment pen were mounted with coloured plastic sheets (three red squares or two blue triangles placed on the sides of the pen). The different colour patterns were used as visual cues to facilitate the association between pen and treatment. Immediately before the testing procedure, the calf was placed in a holding chute positioned at the entrance of the central pen.

### (c) Protocol

Calves were all given one session where they were provided free access to all three pens simultaneously within the apparatus before receiving treatments to avoid a potential effect of novelty.

Calves were disbudded one bud at a time during two separate treatment sessions that took place 24 h (Treatment 1) and 72 h (Treatment 2) after pre-exposure. The two treatments were counterbalanced across the two treatment pens (i.e. if they received Treatment 1 in the pen with blue triangles, they received Treatment 2 in the pen with red squares and vice versa). Hence, all calves were subjected to both procedures: *NSAID* (where they received post-procedural pain control) and *Control* (where they did not). During treatments, calves were kept in the pens for 6 h. See electronic supplementary material for details.

Testing occurred 48, 72 and 96 h after the second treatment (Tests 1, 2 and 3, respectively). The test procedure was similar to pre-exposure: the calves were brought to the apparatus, where the removable gates had been withdrawn. During testing, calves were allowed free access to all three pens until they lay down (for at least 1 min, which ended the session) or after 60 min had passed; they were then returned to their home pen.

### (d) Statistical analysis

Time spent in each of the treatment pens (s) during testing was analysed with a mixed linear model using the lme4 R package [16,17]. The effect of treatment on which pen calves chose to lie down in was analysed with a *χ*^2^ test. See electronic supplementary material for details.

## 3. Results

Three calves were excluded from the study: two for not fulfilling pre-exposure criteria (see electronic supplementary material) and one for falling sick between the first and second treatment (low milk consumption, diarrhoea and rectal temperature greater than 40°C; this calf later recovered), which resulted in 16 calves in the *meloxicam* group and 15 in the *ketoprofen* group.

During pre-exposure, calves did not differ in the time spent in the two different pens (paired *t*-test, *t*_30_ = −0.3, *p* = 0.7).

**Figure 2.**
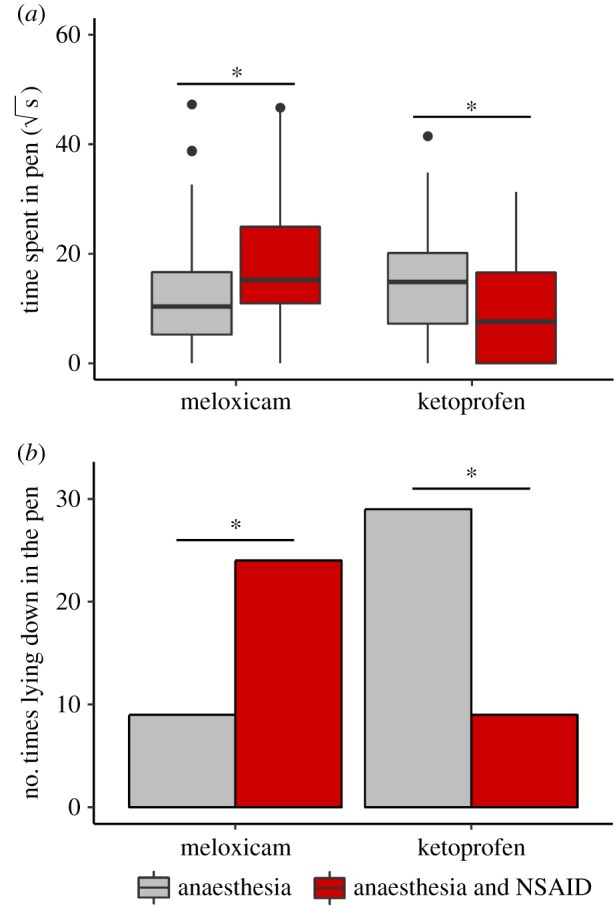
The time (√s) that calves spent in test pens (*a*) and the pen in which calves eventually lay down (*b*) during test sessions, shown in relation to the conditioning treatments. Treatments were *Control* (sedation, local anaesthesia and hot-iron disbudding) and *NSAID* (sedation, local anaesthesia, NSAID and hot-iron disbudding); calves received either meloxicam or ketoprofen as the NSAID. An asterisk (*) indicates a significant difference (*p* < 0.05). (Online version in colour.)

After conditioning, time spent in the pen was not affected by test session number, order of treatment, colour of treatment pen and side of the first bud disbudded (*t*_1,152_ = 0.4, *p* = 0.7; *t*_1,26_ = 0.6, *p* = 0.5; *t*_1,26_ = 0.5, *p* = 0.6; *t*_1,26_ = −0.3, *p* = 0.8, respectively) but there was an interaction between treatment received in the pen (Control or NSAID) and the NSAID used (meloxicam or ketoprofen) (*t*_1,152_ = 4.4, *p* < 0.001). We, therefore, repeated the test of treatment separately for the two NSAIDs. In the meloxicam group, calves spent more time in the pen where they had received the NSAID compared with the control pen where no NSAID was provided (*t*_1,78_ = 2.6, *p* = 0.01). In the ketoprofen group, calves spent less time in the NSAID pen compared with the control pen (*t*_1,73_ = −3.7, *p* < 0.001, figure 2*a*).

Out of the 93 tests (31 calves × 3 tests), there were five in which calves did not lie down within the 60 min session (distributed among three calves: two in the meloxicam group, one in ketoprofen). Among the remaining tests, calves in the meloxicam group lay down more frequently in the NSAID pen (*χ*^2^ = 6.8, *p* = 0.009), and calves in the ketoprofen group showed the opposite pattern (*χ*^2^ = 10.5, *p* = 0.001, figure 2*b*).

## 4. Discussion

Aversion to the pen where calves had been disbudded varied depending upon the type of NSAID used. As predicted, calves that received meloxicam showed more aversion to the pen where they had been disbudded without the NSAID. Surprisingly, calves that had received ketoprofen avoided the pen where they had received the NSAID relative to the pen with the control treatment. We suggest that this difference in place conditioning might be explained by the differences in the duration of action of the two drugs. Meloxicam has an elimination half-life of approximately 25 h [18], whereas ketoprofen's half-life is about 3 h [19]. This difference likely means that only meloxicam was effective in preventing post-procedural pain for the entire 6 h conditioning period while calves were kept in the treatment pen. The shorter duration of action of ketoprofen means that the calves were likely experiencing pain at the end of the conditioning session. This is in accordance with studies reporting a rise in plasma cortisol 3–4 h after disbudding in calves provided with ketoprofen [20,21].

We hypothesize that the aversion to the ketoprofen pen was related to this timing of analgesic effects and its importance on place conditioning. In the control condition, calves only received a local block. The effect of this block likely waned at about the time calves recovered from the xylazine sedation, meaning that during the majority of the conditioning session the post-procedural, inflammatory pain was untreated. In the ketoprofen condition calves probably received some analgesic benefit from the drug, but the protective effect likely diminished over the course of the session. For example, calves given a local block had higher plasma cortisol 4 h after disbudding (i.e. after the local block is expected to have worn off) compared with control calves that had not received a local block [15]. There is evidence that a worsening pain trajectory is especially aversive. Humans focus more on the final moments of an event in their recall of painful experiences, rather than the total duration of pain [22,23]. We encourage researchers to consider the time course of painful experiences when assessing aversion using place conditioning paradigms. Calves provided with ketoprofen 2 h before disbudding and again 2 and 7 h after the procedure showed fewer behavioural events indicating pain (including ear-flicks and head-shakes) in the 24 h after disbudding compared with control calves [24]. Thus, future work could attempt more frequent treatments with ketoprofen, or the use of other drugs with different durations of analgesia, to more directly test the hypothesis that conditioned place avoidance varies according to the time trajectory of the pain.

Ketoprofen can have a number of negative effects (e.g. on the digestive system, central nervous system, cardiovascular system, etc.) when administered to humans [25–28]. We did not observe any evidence of such effects, and consider them unlikely following a single dose, but these direct effects are impossible to rule out in the current study.

As our experiment did not include a sham treatment in which calves were not disbudded, our results do not provide evidence that meloxicam eliminates the post-procedural pain associated with disbudding, only that it makes the memory of the procedure less aversive. We also note that the post-procedural pain associated with disbudding is thought to exceed the 6 h duration of focus in this study [15,29,30]. Conditioned place avoidance (as well as other measures of affective state, see [14,31,32] for details) could be used to better assess the time course of this experience by conducting separate conditioning trials at different times relative to the procedure and analgesic treatments.

Although we did not observe an effect of treatment order, it is possible that our sample size did not allow us to detect the impact of the lingering pain from the first treatment during the second disbudding. There are other factors worthy of attention regarding pain associated with horn removal, including the method used (e.g. amputation [20], caustic paste [1], clove oil [6]) and calf age at the time of the procedure [33]. Place conditioning seems to be an appropriate paradigm to study aspects of the affective component of pain in calves and other animals; we recommend adopting similar approaches to develop more effective methods of reducing pain in animals.

## 5. Conclusion

Meloxicam treatment made hot-iron disbudding less aversive to calves during the 6 h following the procedure, but ketoprofen treatment made the experience more aversive. Further research is needed to determine the number and timing of analgesic treatments required to control post-procedural pain when disbudding. We recommend the use of place conditioning to explore the affective component of pain in animals.

## Supplementary Material

Supplementary material – Detailed methods

## Supplementary Material

Data set

## Supplementary Material

R code

## Data accessibility

Data and R code are freely accessible in the electronic supplementary material.
