# Supplementary material for: Assessing the affective component of pain, and the efficacy of pain control, using conditioned place aversion in calves
Source: Biol Lett. 2019 Oct 30;15(10):20190642. doi: 10.1098/rsbl.2019.0642 (PMC6832189; doi:10.1098/rsbl.2019.0642)
Supplement: Supplementary material – Detailed methods [file rsbl20190642supp1.docx]

**Supplementary material – Detailed methods – Biology Letters**

**Assessing the affective component of pain, and the efficacy of pain control, using conditioned place aversion in calves**

Thomas Ede^1^, Marina A. G. von Keyserlingk^1^ and Daniel M. Weary^1^*

^1^Animal Welfare Program, Faculty of Land and Food Systems, University of British Columbia, Vancouver, B.C., Canada

*Correspondence to [dan.weary@ubc.ca](mailto:dan.weary@ubc.ca)

**Sample size**

We first aimed to enroll 36 calves (n = 18 per treatment group; double the sample size of 9 calves per treatment used in our previous study [1]). Due to time and calf availability constraints we were able to enrol only 34 calves; of these three were excluded, two for not fulfilling pre-exposure criteria (see ‘Pre exposure’ section) and one for falling sick between the first and second treatment (low milk consumption, diarrhea and rectal temperature > 40 °C)

**Housing**

Calves were housed in groups of 8, starting at 7 d of age, in pens measuring 4.9 x 7.3 m, bedded with sawdust. All calves had *ad libitum* access to hay and water and were provided 12 L of whole milk per day through an automatic milk feeder (CF 1000 CS Combi; DeLaval Inc., Sweden). At the time of testing, calves were brought individually from their home pen to the experimental apparatus at about 11 am.

**Pre exposure**

Calves were brought from their home pen and placed within the holding chute and provided a small milk reward (approximately 0.2 L). While in the chute, they were injected subcutaneously on the right side of the rump with saline (0.9% Sodium chloride, Hospira, Montreal, Canada) at 0.01 mL/kg; a volume identical to what they would receive when injected with xylazine during the treatment phase. The gate into the central pen was then opened, allowing calves to enter the apparatus. This pre-exposure session lasted 15 min, after which calves were gently brought back to their home pen. Calves that failed to explore all three pens during this pre-exposure session were excluded from the experiment (n = 2).

**Treatments**

*NSAID* procedure: calves were brought from their home pen to the chute where they received a 0.2 L of milk reward before being injected with 0.2 mg/kg of xylazine (SC, right rump, Rompun 20 mg/mL, Bayer, Leverkusen, Germany). Immediately following the injection, calves were led and locked inside one of the treatment pens; the handler waited approximately 10 min for the calf to show signs of sedation (i.e. recumbency with eyeball rotation, see Ede et al. [2]). Once sedation was achieved, calves were injected with 5 mL of lidocaine (Lido-2 [lidocaine 2%, Epinephrine 1:100,000], Rafter8, Calgary, Canada) in the lateral canthus of one eye with a 0.9 mm x 25 mm needle (8881251782, Covidien, Dublin, Ireland). Each calf then received one of the NSAID treatments according to recommended use (meloxicam group: SC, neck, 0.5 mg/kg, Metacam, 20 mg/mL, Boehringer Ingelheim, Burlington, Ontario, Canada; ketoprofen group: SC, neck, 3 mg/kg, Anafen, 100 mg/mL, Boehringer Ingelheim, Burlington, Ontario, Canada). 10 min later, the horn bud on the side of the lidocaine injection was shaved, tested for pain-reflex with a needle-prick and disbudded by placing a 500°C hot-iron (X30, 1.3 cm tip, Rhinehart, Spencerville, IN, USA) over the horn bud for approximately 15 s. The calf was then positioned in sternal recumbency and left in the treatment pen for the next 6 h before being returned to their home pen.

*Control* procedure: The procedure was identical to that described above for the NSAID procedure but instead of receiving an NSAID, calves were injected with saline (0.9% Sodium chloride, Hospira, Montreal, Canada, with a volume similar to the NSAID injection).

Order of treatment (Control or NSAID first), treatment pen associated with NSAID (Red squares or Blue triangles), first horn disbudded (Left or Right) and pre-exposure preference (i.e. treatment associated with the pen in which calves spent the most time during pre-exposure) were balanced across treatments.

**Statistical analysis**

The model’s fixed factors were the treatment received in the pen (Control or NSAID, 1df), which NSAID was used (meloxicam or ketoprofen, 1 df), their interaction (1df), the test session number (1, 2 or 3, 1 df), which treatment was received first (Control or NSAID, 1df), which treatment pen was associated with the NSAID procedure (blue triangles or red squares, 1df) and which bud was first disbudded (Left or Right, 1 df). Calf was considered a random factor (n = 31). The total number of pseudo-repeated observations of time spent in pens was 186 (31 calves x 3 sessions x 2 pens). Normality and homoscedasticity of residuals were confirmed graphically after square root transformation. P-values were calculated with Satterthwaite’s approximations using the lmerTest R package [3].

**References**

1. Ede T, Lecorps B, Keyserlingk MAG von, Weary DM. 2019 Calf aversion to hot-iron disbudding. *Sci. Rep.* **9**, 5344. (doi:10.1038/s41598-019-41798-7)

2. Ede T, von Keyserlingk MAG, Weary DM. 2019 Efficacy of xylazine in neonatal calves via different routes of administration. *Vet. J.* **247**, 57–60. (doi:10.1016/j.tvjl.2019.02.012)

3. Kuznetsova A, Brockhoff PB, Christensen HB. 2017 lmerTest package: tests in linear mixed effects models. *J. Stat. Softw.* **82**. (doi:10.18637/jss.v082.i13)
